# Notes

**Published:** 1898-01

**Authors:** 


### Notes.

A new medical journal, published in Philadelphia under the editorship of Dr. Geo. M. Gould, made its appearance the first of the year.

Mr. Jonathan Hutchinson, F.R.S., has intimated his willingness to found an educational museum in his native town of Selby, Yorkshire.

Mr. Frederick Treves has resigned the Lectureship on Surgery at the London Hospital, and has been succeeded by Mr. Mansell Moullin.

Governor Adams, of Colorado, has signed the bill prohibiting the sale of cocaine without a written prescription of a licensed physician or dentist.

A Louisville physician at present practising in the Klondike gold region is reported to be making a fortune by selling capsules of quinine at three dollars apiece.

The ninth International Congress of Hygiene and Demography, under the patronage of His Majesty the King Alphonso XIII., and the Queen Regent, will convene at Madrid, in April.

Among the institutions in which Atlanta takes pride, is the Holmes' Sanatorium, which has done and is doing splendid work for the afflicted, and adding to the name and character of the city abroad. The inspiration of this remark is a personal knowledge of its work and excellence.—*Atlanta Presbyterian.*

The McIntosh Battery and Optical Company of Chicago, have issued a complete catalogue of their goods combined with an electro-therapeutic. Every one who has not a work on this subject would do well to possess himself of this catalogue, as it contains about all that he can need of practical information on the subject.

At the recent International Congress on Leprosy held in Berlin, Professor Virchow was elected president. Dr. Hansen, of Norway, in his address emphasized the necessity of isolating lepers, a measure which he said was of as much interest to the patients as to the community at large. In Dr. Hansen's opinion the efforts of Unna, Carasquilla and Danielson to cure leprosy by means of drugs and the serum treatment have been quite ineffectual, claiming that the disease decreases only under the influence of strict isolation and increases again when isolation ceases to be enforced. He also claimed that the hypothesis that leprosy might be due to hereditary influence was erroneous.—*Medical Age.*

### A LONG RANGE "TOUCH."

"Mr. Campbell, caterer for one of the 'messes' at the University of Virginia has recently been robbed of $200 and a medical student *of New Jersey* has been arrested as being the thief."—*Ex.*

### FIRST AID TO THE INJURED.

There was a young lady named Cheedle,
Who, at church, sat down on a needle;
Though deeply imbedded
It was luckily threaded
And was promptly removed by the beadle.

### A NEW SURGICAL ACHIEVEMENT.

From Switzerland comes the report that Dr. Carl Schlatter, of Zurich, has made a complete removal of the stomach. This operation has excited a good deal of wonder in the lay mind, but with the triumphs of gastro-intestinal anastomosis already before us, it is certainly no new idea that the stomach is not a vital or even a necessary organ. Our knowledge of intestinal digestion should certainly be sufficient to make the stomach a physiological superfluity from the standpoint of nutrition. It will be interesting to note, when the time arrives, to what extent Nature will adapt the end of the intestinal tube to the form and function of the removed organ—to observe, in fact, how far she will condone surgical audacity.—*Medical Age.*

### SURGICAL HINTS.

In the treatment of abscess, free drainage is far more important than the use of chemical antiseptics.

In ligating vessels the fine ligature is best so long as it is strong, for the knot is less liable to slip.

Cancer of the breast occurring during pregnancy or lactation is particularly malignant, and is of very rapid growth.

Do not drain a healing cavity for too long a time. Your drain may be acting as a seton, actually keeping up the suppuration.

A tumor which having existed for a long time suddenly begins to grow, should be regarded with the gravest suspicion. It is probably malignant.

In draining spaces whose walls are flaccid and tend to fall together, gause does very well. When there is a true cavity, however, a tube will usually be more efficient.

In diagnosing abdominal tumors it is always best to clear the patient's bowels thoroughly, and then palpate in full surgical auæsthesia before venturing a positive opinion.

When chronic intestinal obstruction is caused by carcinoma, wherever located, there is, practically, always an intermittent pain, of a paroxysmal nature, situated in the reigon of the umbilicus.

Dermoid cysts at the outer angle of the brow are often taken for lipomata or for wens. These dermoid cysts are not easy to remove, for they are very firmly attached to the bone, often, too, by a wide base.

If you are about to examine a septic case or one where you suspect syphilis, wash your hands in vinegar or dilute acetic acid, and you will soon discover by the smarting any little scratches or abrasions in your skin which might become the starting points of infection.

When you wish to pack a large cavity with gause, a good way is to fit a single layer or sheet of the material all round against the walls of the space, allowing the edge of the gause to remain outside. You then have a gause bag lining the cavity. Into this bag you may pack as much gause as may be necessary for pressure, or for thorough absorption of the discharges. When the time comes for removing the packings you will find that by first removing the inner filling of the gause bag, and last of all the bag itself, there will be comparatively little pain, little hemorrhage, and little derangement of the tissues.—*International Journal of Surgery.*

